# Depressive disorders and children with chronic illness

**DOI:** 10.1192/j.eurpsy.2021.584

**Published:** 2021-08-13

**Authors:** O. Mihailov, I.D. Rădulescu, R. Mihailov, A. Ciubară

**Affiliations:** 1 Pediatric Pneumology, Hospital of Pneumophysiology “Saint Spiridon”Galati, Galati, Romania; 2 Psychiatrist, “Elisabeta Doamna” Psychiatric Hospital, Galati, Romania; 3 Surgery, “Dunarea de Jos” University of Galati, Faculty of Medicine and Pharmacy, Galati, Romania; 4 Md, Ph.d., Hab. Professor, Faculty of Medicine and Pharmacy, University “Dunarea de Jos” Head of Psychiatry Department, Senior Psychiatrist at ”Elisabeta Doamna” Hospital, Galati, Romania

**Keywords:** tuberculosis, children, depression

## Abstract

**Introduction:**

When depression is comorbid with tuberculosis, it will lead to decreased quality of life, lack of adherence to anti-Tb drugs, progression to MDRTB and will end in death with mortality from the disease.

**Objectives:**

We aimed to study the association of Tuberculosis and depressive disorders in children aged 7-18 years compared to non-tuberculosis diseases and their correlation. We hypothesized that depression will be significantly more common in patients with tuberculosis than in non-TB patients, who served as a control.

**Methods:**

A prospective observational case-type study for a period of 2 years, 2018-2020. The patients included in the study are patients diagnosed and treated in the Child Pneumology Department of the Pneumoftiziology Hospital “Sfantul Spiridon” Galati and in the TB Dispensaries in Galati County divided into the study group consisting of patients diagnosed with Tuberculosis and the control group of patients without a diagnosis of Tuberculosis or other previous chronic disease. For the diagnosis of depression in the case of the two groups, we used the CDI questionnaire (Depression Inventory for children).

**Results:**

Out of 100 children with TB, 68% had depression compared to the control group, which showed that only 9% had depression.

**Conclusions:**

Depression can affect all parts of a child’s life, including behavior, appetite, energy levels, sleep patterns, relationships, and academic performance. We observe a wide range of symptoms in the group of children with tuberculosis compared to the control group.

